# Insights into the Promising Prospect of G Protein and GPCR-Mediated Signaling in Neuropathophysiology and Its Therapeutic Regulation

**DOI:** 10.1155/2022/8425640

**Published:** 2022-09-21

**Authors:** Md. Mominur Rahman, Md. Rezaul Islam, Sadia Afsana Mim, Nasrin Sultana, Dinesh Kumar Chellappan, Kamal Dua, Mohammad Amjad Kamal, Rohit Sharma, Talha Bin Emran

**Affiliations:** ^1^Department of Pharmacy, Faculty of Allied Health Sciences, Daffodil International University, Dhaka 1207, Bangladesh; ^2^Department of Life Sciences, School of Pharmacy, International Medical University, Bukit Jalil, 57000 Kuala Lumpur, Malaysia; ^3^Discipline of Pharmacy, Graduate School of Health, University of Technology Sydney, Ultimo, NSW 2007, Australia; ^4^Institutes for Systems Genetics, Frontiers Science Center for Disease-Related Molecular Network, West China Hospital, Sichuan University, Chengdu, 610041 Sichuan, China; ^5^King Fahd Medical Research Center, King Abdulaziz University, Saudi Arabia; ^6^Enzymoics, Novel Global Community Educational Foundation, Australia; ^7^Department of Rasa Shastra and Bhaishajya Kalpana, Faculty of Ayurveda, Institute of Medical Sciences, Banaras Hindu University, Varanasi, 221005 Uttar Pradesh, India; ^8^Department of Pharmacy, BGC Trust University Bangladesh, Chittagong 4381, Bangladesh

## Abstract

G protein-coupled receptors (GPCRs) are intricately involved in the conversion of extracellular feedback to intracellular responses. These specialized receptors possess a crucial role in neurological and psychiatric disorders. Most nonsensory GPCRs are active in almost 90% of complex brain functions. At the time of receptor phosphorylation, a GPCR pathway is essentially activated through a G protein signaling mechanism via a G protein-coupled receptor kinase (GRK). Dopamine, an important neurotransmitter, is primarily involved in the pathophysiology of several CNS disorders; for instance, bipolar disorder, schizophrenia, Parkinson's disease, and ADHD. Since dopamine, acetylcholine, and glutamate are potent neuropharmacological targets, dopamine itself has potential therapeutic effects in several CNS disorders. GPCRs essentially regulate brain functions by modulating downstream signaling pathways. GPR6, GPR52, and GPR8 are termed orphan GPCRs because they colocalize with dopamine D1 and D2 receptors in neurons of the basal ganglia, either alone or with both receptors. Among the orphan GPCRs, the GPR52 is recognized for being an effective psychiatric receptor. Various antipsychotics like aripiprazole and quetiapine mainly target GPCRs to exert their actions. One of the most important parts of signal transduction is the regulation of G protein signaling (RGS). These substances inhibit the activation of the G protein that initiates GPCR signaling. Developing a combination of RGS inhibitors with GPCR agonists may prove to have promising therapeutic potential. Indeed, several recent studies have suggested that GPCRs represent potentially valuable therapeutic targets for various psychiatric disorders. Molecular biology and genetically modified animal model studies recommend that these enriched GPCRs may also act as potential therapeutic psychoreceptors. Neurotransmitter and neuropeptide GPCR malfunction in the frontal cortex and limbic-related regions, including the hippocampus, hypothalamus, and brainstem, is likely responsible for the complex clinical picture that includes cognitive, perceptual, emotional, and motor symptoms. G protein and GPCR-mediated signaling play a critical role in developing new treatment options for mental health issues, and this study is aimed at offering a thorough picture of that involvement. For patients who are resistant to current therapies, the development of new drugs that target GPCR signaling cascades remains an interesting possibility. These discoveries might serve as a fresh foundation for the creation of creative methods for pharmacologically useful modulation of GPCR function.

## 1. Introduction

Several genetic alterations and mutations have been recognized as hazardous factors in the pathogenesis of mental illnesses, like schizophrenia, bipolar disorder, and depression. However, although there have been substantial advancements in strategies to aid develop potent drugs as therapeutics for psychiatric disorders, currently, there is a gap in bridging treatment modalities with disease progression. This provides a continuing impetus to develop effective treatment options for such health conditions [1]. In the face of multiple complex genetic and environmental risk factor challenges faced when designing a targeted novel treatment for such pathological conditions, multiacting receptor-targeted antipsychotics (MARTA) have been considered as antipsychiatric drugs [2, 3, 4]. Two kinds of receptors that have different synaptic transmission modalities are how neurotransmitters carry out their tasks. Fast synaptic transmission is induced by ligand-gated ion channels, which are made up of ionotropic receptors. Contrarily, metabotropic receptors are made up of GPCRs that bind to neurotransmitters and activate intracellular signaling pathways to reduce synaptic transmission and induce the gene expression required for antipsychotic effects [5, 6]. Notably, it is recognized that the majority of neuropharmacological medications control GPCR activity in the central nervous system (CNS) [7, 8, 9].

**Figure 1:**
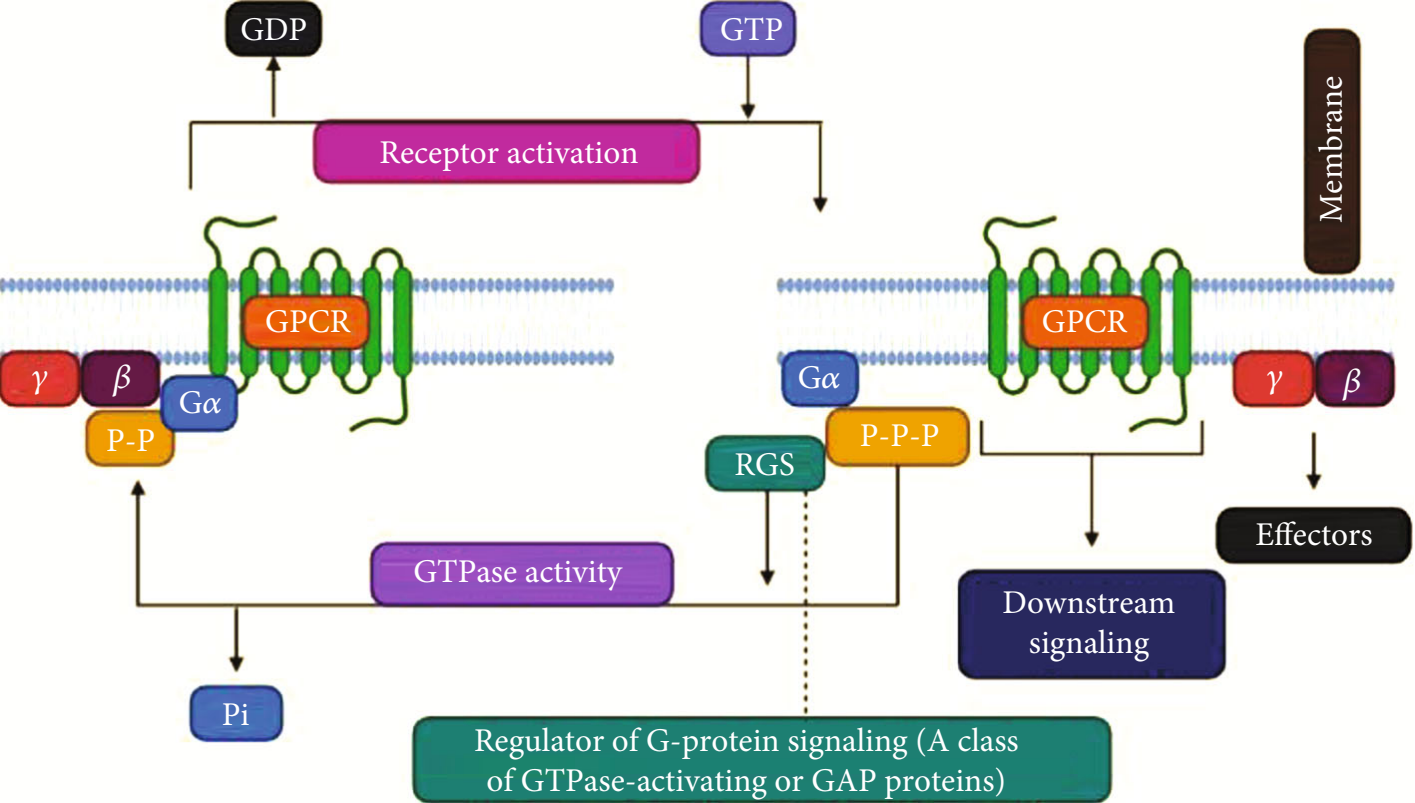
Heterotrimeric G protein signaling with RGS regulation. GPCR activation, either due to agonist binding or constitutive activity, causes downstream signaling through both the *α* and *βγ* subunits. Various antidepressants modulate this process directly (e.g., buspirone) or indirectly (e.g., SSRIs). RGS proteins interact with active G*α* and accelerate its GTPase activity, facilitating a return to the GDP-bound inactive state. Preclinical models suggest that direct manipulation of the RGS or G proteins can affect antidepressant response.

G protein-coupled receptors are 7-transmembrane domain proteins that form a multiprotein complex with members of an intracellular family of heterotrimeric G proteins comprised of a G*α* subunit and a *βγ* dimer (Figure 1). There are several members of the G*α* family that couple to different cohorts of effectors in the cell. GPCRs are the largest transmembrane proteins in the human genome. Half of such GPCRs possess sensory function, whereas the remaining half possess nonsensory function [10]. The nonsensory GPCRs regulate communications between various cells following activation via ligands like amino acids, peptides, amides, and lipid-derived products. Indeed, a vast majority of the currently available drugs work through GPCRs. In addition, GPCRs regulate a wide range of physiological functions, including chemosensory identification, endocrine regulation, and behavioral events [11].

Almost 90% of the nonsensory GPCRs exert their roles in both normal and complex brain functions and in regulating CNS physiology [12]. In this light, GPCRs have formed a primary target for antipsychotics like aripiprazole, olanzapine, and quetiapine. Some 367 receptors have been reported to contribute towards the nonsensory GPCRs [13]. Additionally, classical GPCRs, namely, dopamine, acetylcholine, and glutamate, have long been neuropharmacological targets in neurological/neuropsychiatric drug development. As a consequence, such protein binding sites can be considered to mediate potential therapeutic effects in CNS disorders [14], and supporting this, a substantial proportion of current medications that are related to neuropharmacology act via GPCR-pathways within the central nervous system [15]. GPCR pathways are activated when they receive signals from the G protein. Receptor phosphorylation through G protein-coupled receptor kinases (GRKs) provides the main impetus for this type of signaling pathway [16, 17]. Antipsychotic drugs, mediated through dopamine receptors, are widely employed in CNS disorders, and the dopaminergic system is additionally important and provides a drug target in bipolar disorder, schizophrenia, Parkinson's disease (PD), and ADHD [18, 19, 20]. Dopamine essentially acts on GPCRs with the potential ability to activate the G protein through either the Gi, Gs, or Gq pathways [21]. Mitochondrial complexes [22] and altered gene expressions [23] have been reported in such signaling cascades. Oxidative stress is a common feature too, due to the oxidative metabolism of dopamine [24], which may additionally induce glutathione [25].

Furthermore, glutathione's precursor N-acetyl-cysteine is a potent antioxidant and has, likewise, been extensively investigated in psychiatric disorders [26–28]. Mental health diseases have yet to benefit from the present molecular medicine revolution, which has had an immediate influence on other areas of medicine. Research into the genesis and pathophysiology of complicated mental diseases is hampered by more than simply the CNS's intrinsic complexity. These include an absence of a clearly defined pathology, limited access to tissues, and the humbling realization that behavior is more complicated than the sum of its parts [29]. Appropriate diagnosis is one of the most challenging issues in the development of a novel treatment approach when it comes to psychiatric disorders with limited understanding of their mechanism impacting cognition, behavior, and emotion. In this regard, an altered psychological function is strongly linked to psychiatric conditions. Consequently, GPCRs have been widely reported to possess huge potential with respect to potentially developing novel drugs or treatment approaches for psychiatric disorders [8, 30].

Therefore, this review is additionally aimed at investigating the pharmacokinetics, pharmacodynamics, and the significance GPCRs for developing novel treatment strategies for psychiatric disorders. The involvement of G protein-coupled receptors (GPCRs) in the development and treatment of serious mental diseases has nevertheless made significant progress in our knowledge. In this position essay, we analyze and integrate the knowledge that is presently available and discusses how it might be used to generate better drugs strategically. Due to space constraints, this review's analysis has been limited to the most important mental illnesses and the most important GPCR families that have been implicated. Wherever possible, we have used reviews in place of source materials.

## 2. GPCRs in Central Nervous System

Humans and mice each contain 367 nonodorant GPCR superfamily members, with 343 common receptors, based on thorough analysis of public databases for mouse and human genome sequences, according to the study. There are 83 mouse and 26 human GPCRs that have not yet been discovered. Unexpected levels of orthology are found when GPCRs from the two species are directly compared. The survival of these molecules over time disproves functional redundancy among closely comparable receptors [31]. Nonodorant GPCRs are highly expressed in the central nervous system, notably in the brain, with orphan receptors accounting for one-fourth of those found [32]. Nonodorant GPCRs make up around 80% of the 353 nonodorant GPCRs that are expressed in the mouse CNS. It has been shown by Komatsu that the CNS expresses considerable and selective levels of mRNA for six clusters of GPCRs, which collectively account for approximately 40% of the 322 nonodorant GPCRs discovered in mice. These six clusters are rich in neuropharmacological targets like dopamine, serotonin, acetylcholine, and glutamate which all have receptors for these chemicals in these clusters. The nonodorant GPCRs that are peculiar to the brain have a lot of potential as CNS medication targets, according to these findings [33].

## 3. GPCRs and Mood Disorders

### 3.1. Noradrenergic Receptors and Mood Disturbances with G Protein-Coupled Receptors

The distinctive primary synaptic components responsible for influencing emotional problems include the noradrenergic framework. After investigating the structure of peripheral blood cells, several critical assessments can be made with regard to the role of noradrenergic receptors in emotional disorders.

#### 3.1.1. Studies on Peripheral Blood Cells

*(1) Alpha2-Adrenergic Receptors*. Several studies have reported alterations in blood platelets and alpha2-adrenergic receptor (alpha2-AR) mechanisms in subjects with mood disorders. In addition, it has also been observed that there were minimal changes in the receptor numbers in studies conducted with yohimbine-alkaloid radio ligands; one of the first radioligands developed to allow selective labeling of alpha2-ARs. Studies conducted with partial or complete agonists demonstrated wide variations in their activities, especially in weaker patients [34]. However, these findings highlighted several uncertainties, as the ligands involved here were bound to noradrenergic imidazoline receptors that were present on platelets [35, 36, 37]. Considering these findings, the sensitivity of alpha2 receptors seems inconclusive. This subject has also revealed the intricacies of assessments involving the bipolar issue. In addition, it was observed that the thickness of the alpha2-AR was enhanced in the platelets patients' who had bipolar disorder [38]

*(2) Beta2-Adrenergic Receptors*. Studies on the quantities of beta2-adrenergic receptors in peripheral cells, however, have shown inconsistent outcomes, with one finding being that depressed individuals' leukocytes have lower levels of beta-AR-stimulated AC activity [39, 40, 41, 42]. It has been postulated that this could be due to dysfunction of the receptor/Gs/AC complex. To date, it remains unclear which abnormalities in peripheral cells are identified with the pathophysiology of mood disorders and are associated with nonspecific stress or homeostatic mechanisms through both alpha2 and beta2 observations [43–45].

#### 3.1.2. Impacts of Antidepressants and Mood Stabilizers

*(1) Alpha-Adrenergic Receptors*. Antidepressants have been demonstrated to lower alpha2-ARs in animal tissues, whereas alpha1-ARs have been shown to be increased [46]. Interestingly, prolonged lithium treatment has been found to reduce alpha2-AR-mediated behavioral consequences [47].

*(2) Beta-Adrenergic Receptors*. The majority of studies have discovered a predictable pattern of beta-AR downregulation in response to long-term antidepressant or electroconvulsive therapy treatment (ECT) [48, 49, 50, 51]. In the brain, long-term use of desipramine has been linked to an uncoupling of beta-AR and Gs [52, 51]. Receptor downregulation and receptor-G protein uncoupling both have been reported responsible for reduced downstream of cAMP signaling [53]. Moreover, desipramine prevents the dissolution of the beta-AR high-affinity complex, which prevents adenylate cyclase from being activated downstream [54]. Lithium has been the subject of critical inquiries focusing on the results of evacuation stabilizers. There have been several studies assessing the effect of lithium on the actual adrenergic receptor in rodent cerebrums. However, an obstacle to beta-AR was that cAMP [55] was destroyed. Moreover, lithium does not avoid stimulant-instigated beta-AR downregulation [56].

#### 3.1.3. Receptor Polymorphisms

Whereas there have been no reported associations between alpha2-AR polymorphisms and suicide or depression [57, 58], one study identified a nonsignificant link between a beta1-AR polymorphism, G1165C, and an increased antidepressant efficacy rate in depressed patients [59]. In recent research, the outcomes of yohimbine infusion on people with an alpha2C-adrenoreceptor subtype an-frame deletion (alpha2CDel322-325) [60] was studied. There was more whole-body noradrenaline spillover in homozygotes for the alpha2CDel322-325 polymorphism while they were at rest than in heterozygous individuals. In homozygotes, the effects of yohimbine were more pronounced and persisted longer than in the other groups. Because catecholaminergic responses have a similar impact on mood disorders, this receptor polymorphism is being studied more thoroughly in this area.

### 3.2. Serotonergic Receptors Coupled with G Protein and Mood Disturbances

The efficacy of serotonergic-mediated medications in the management of depression has been a driving force behind the idea that serotonin plays a crucial role in sadness and mood disorders. In this regard, the “serotonin hypothesis” of clinical depression is now over 50 years old and, in a nutshell, proposes that reduced serotonin-mediated signaling has a pivotal role in the pathophysiology that culminates in depression. This view is based on the adverse depressive actions of amine depleting drugs exemplified by reserpine, the antidepressant properties of tricyclic antidepressant and monoamine oxidase inhibitor drugs that augment serotonin and other monoamines within the synapse, and extensive studies showing aberrant serotonergic signaling in patients with sadness and mood disorders. Parallel information for the bipolar disorder is less extensive but is growing. Further evidence that serotonin plays a role in such dispositions comes from the discovery of receptor polymorphisms linked to illness susceptibility and treatment response in specific regions of the brain.

#### 3.2.1. Impacts of Antidepressants and Mood Stabilizers

*(1) Receptors of 5HT1A*. During long-term antidepressant therapy, serotonin transmission increases in the hippocampus, leading to desensitization of dorsal raphe somatodendritic 5HT1A autoreceptors and support for 5HT1A receptor function in mood disorders [61]. In patients with bipolar disorder, 5HT1A receptor impairments can be mitigated by a stable lithium treatment [62]. A consistent energizer therapy has also been established to enhance the uncoupling of the 5HT1A receptor-G protein [63].

*(2) Receptors of 5HT2*. Antidepressants have been shown to decrease the cortex-authoritative 5HT2A receptor functions [64]. The evidence for the part of the 5HT2A receptor in the disposal stabilizer operation instrument is significantly less clear. Blended results have been provided by exploring the 5HT2A receptors after a consistent lithium association. Lithium induces a typical activity within the cutoff retention of the 5HT2 receptor with the most grounded hippocampal confirmation [62] at the same time when platelets were affected in bipolar sufferers, thereby, limiting the upward thrust of lithium inside the 5HT2A receptors [65].

#### 3.2.2. Polymorphisms of Receptors

*(1) Receptors for 5HT1A and 5HT1B*. Terock et al. and Han et al. [65, 66] have reported that the C-1019G 5HT1A polymorphism is related to a significant self-destruction of the 5HT1A agonist stimulant response to flibanserin. A relationship between the stimulant response and both this polymorphism [67, 68] and Gly272Asp [68] has been revealed in a number of studies. Albert et al. [69] found that the hereditary impact of the 5HT1A receptor-encoding quality of HTR1A and the serotonin carrier encoding quality of SLC6A4 impacts the clinical results of patients treated with citalopram. It has been reported that the G861C locus of the 5HTR1B quality is implicated with distress, yet not with bipolar disorder [70]; albeit, no such association was found in other investigations [66].

### 3.3. Dopaminergic Receptors with G Protein-Coupled and Mood Disorders

Dopamine has long been a focus of schizophrenia-related research, but its role in mood disorders has gotten far less attention. Despite this, mounting data suggests that dopamine plays an important role in a wide range of illnesses. Goal-directed behavior is influenced by the dopamine systems in the midbrain and mesolimbic systems, which are linked to motor activity, motivation, and reward pathways respectively. All of these functions can be substantially damaged in states of depression and mania. Pharmacological therapies for mood disorders have been demonstrated to ameliorate mood symptoms, showing that G protein-coupled dopamine receptors play an important role in the development of mood disorders.

#### 3.3.1. Polymorphisms of Receptors

*(1) Receptor D1*. Whereas various investigations have failed to see any bipolar changes in the D1 receptor gene [71, 72], a connection between polymorphism of the D1 receptor A48G and bipolar disease has been observed [73, 74].

*(2) Receptor D2*. Different research has sought to find a link between polymorphism of the D2 receptor and bipolar illness; however, such a link has not been discovered. Nevertheless, two evaluations have reported cautious, but optimistic outcomes. Overs et al. [75] investigated and reported a critical connection between the D2 receptor and bipolar disorder in an European multicenter examination. Han chinese patients with a bipolar exacerbation was not underlined when attempted in Caucasians. Kulmala et al. [76] saw a relationship with a D2 receptor polymorphism recommending a potential race-express threat issue.

*(3) Receptor D3*. A relationship between D3 receptor polymorphism and unipolar despondency has been observed in one study [75]. Thus far, examinations have failed to show any evidence for the D3 receptor locus association in bipolar issues.

*(4) Receptor D4*. The possible role of the D4 receptor in misery has been investigated in few studies. A meta-assessment of these reported by Lopez Leon and colleagues found a liberal relationship between the existence of the D4 receptor 48 base pair found to be involved through polymorphism and unipolar weakness at any time, but not in a bipolar problem.

### 3.4. Cholinergic Receptors with G Protein-Coupled and Mood Disturbance

There has long been speculation about the role of the cholinergic framework in bipolar disorder. This was based on experiments showing that cholinergic agonists and antagonists have significant mood and behavioral effects. Most of the examinations detailing openness to cholinergic receptors in disposition issues have been exceptionally backhanded. For instance, REM occurs during particular patterns of sleep, yet its initiation might be set off by cholinergic actions as observed in healthy volunteers. The induction of REM sleep in persons with mood disorders who are not taking medication is more rapid with the administration of arecoline (a cholinergic agonist) (primarily focused on bipolar disorder) [62]. Several antidepressants possess anticholinergic effects, including tricyclic and serotonin reuptake inhibitors, and hence, their useful biological actions can sometimes be adversely affected by sedation, psychomotor and memory impairment, a dry mouth, and blurred vision. Several studies have provided contradicting reports on the impact of lithium on function of the muscarinic receptor. It has been reported [77] that lithium obstructs the super sensitivity of the muscarinic receptor despite influencing the quantity of receptor within restricted areas, suggesting that it works at a postreceptor site [78, 79].

### 3.5. GABAergic Receptors and Mood Disturbances That Are G Protein Coupled

G protein-coupled GABA-B receptors have been demonstrated to be beneficial in the treatment of mood disorders in a modest number of trials. GABA-B agonist baclofen has been demonstrated to cause depression in a tiny percentage of individuals; therefore, discontinuing the medication can help alleviate depression [80]. Lithium, valproic acid, and carbamazepine, among other mood stabilizers, increase GABA-B receptors in the hippocampus when taken on a long-term basis [81, 82].

### 3.6. Glutamatergic Receptors Coupled with G Protein and Mood Disorders

Most neurotransmitter systems are thought to have a threshold for excitation that is regulated by the glutamatergic system, which is the principal excitatory neurotransmitter of the CNS. Recently, research has focused on the possible significance of mood disorders in a possible link between the two. According to current investigations, the glutamatergic system appears to be important in the development and treatment of mental health issues [83–85]. Currently, investigations are jointly evaluating both NMDA [85] and AMPA [65] ionotropic receptors in the treatment of mood disorders. These will not be detailed as our focus is GPCRs; however, there are recent reviews that include a comprehensive discussion of the glutamatergic system's function in mood disorders [83–85].

### 3.7. Receptors of G Protein-Coupled Neuropeptides and Mood Disorders

As a neurotransmitter, neuropeptides are a group of short-chain amino acids. One of the initial interests in biological psychiatry was the role of modified endocrine function in mood disorders [86].

#### 3.7.1. Receptors of Corticotrophin-Releasing Factor (CRF)

Because of its growing importance in neurological illnesses and the numerous unanswered questions it has raised in people and animal models, CRF has become one of the most intensively studied neuropeptides [62, 87, 88]. Anxiolytic and overactivity effects have been demonstrated in preclinical studies employing direct models for antagonists of CRF receptors, in particular for subtype CRF receptor-1 [89]. These findings led to the open trial of R121919, a CRF receptor-1 antagonist, in 24 individuals with depression [90]. Initial results have been promising, and further clinical trial results are awaited. At least two genetic linkage and association studies, however, have found no evidence linking CRF polymorphisms to bipolar disorder [91–93].

## 4. Schizophrenia and GPCRs

### 4.1. Dopaminergic Receptors Coupled to G Protein and Schizophrenia

Despite the fact that the underlying pathogenesis of schizophrenia is clearly highly complex, the dopamine hypothesis and its various revisions provide a valuable and scientifically based explanation for the disease's psychotic symptoms (particularly hallucinations and delusions). Dopamine dysregulation in emotional processing areas like the amygdala and hyperactivity of dopamine transmission in the mesolimbic areas are hypothesized to be associated with schizophrenia, as is the hypoactivity of dopamine transmission in the prefrontal cortex.

#### 4.1.1. Impacts of Antipsychotic Medications

To begin with, the idea of overactive dopamine was put up due to research showing that conventional antipsychotics have a high D2 affinity and that receptor affinity has a direct correlation to the clinical response [94]. Antipsychotic effectiveness appears to be best around 65% to 70% D2 receptor occupancy, while higher than 80% occupancy greatly raises the incidence of extrapyramidal symptoms (EPS), a well-known adverse effect. PET and SPECT investigations have supported this connection. However, several studies have shown that D2 receptor binding alone is not adequate to explain schizophrenia's physiology. In the first place, despite 70 percent or more receptor occupancy, standard antipsychotics are not helpful for all people with schizophrenia [3]. Second, traditional antipsychotics are only successful in treating about 70% of positive symptoms [4] and have been reported ineffective in treating negative and cognitive symptoms [95].

#### 4.1.2. Receptor Polymorphisms

In the light of the pivotal role of D2 receptors in the pharmacological action of antipsychotics, numerous investigators have looked into the role of D2 receptor polymorphisms in schizophrenia. Several studies, that have included different groups of independent investigators undertaking metaexaminations, have reported a positive relationship between polymorphisms and schizophrenia related to the D2 receptor Ser311Cys [96–99]. Nonetheless, other evaluations have found no connections [99]. A relationship between Ser311Cys and adverse effects was found in one of the investigations [100]. Furthermore, a few further studies have revealed that schizophrenia is linked to a second polymorphism in the D2 receptor, which highlight a specific region, -141C Ins/Del [101, 102]. Various assessments have additionally described assorted other polymorphisms of the D2 receptor, for instance, C957T [103], His313 [104], and Taq1A locus [105]. Several studies have linked the -141C Ins/Del allele to the time or degree of response to antipsychotic drugs, with the -141C Ins allele being associated with a better response; however, other studies have contradicted these findings [60, 104, 106, 107]. The association between treatment effects and the Taq1A locus [108], Ser311Cys polymorphism [68], and His452Tyr polymorphism [107] has been evaluated in a number of studies. The D3 receptor polymorphism, Ser9Gly, is the most frequently analyzed in relation to the D3 receptor, regardless of a lack of schizophrenic symptoms associated with it [109, 110]. The connection between this polymorphism and the response to antipsychotics has been reported in a few studies [111, 112]. There is some distinction from these examinations concerning which subset of indications principally is connected with this polymorphism [113]. The Gly9 allele is reported to be associated with the response to atypical antipsychotics [104]. G-205A [114], G-7685C [111], and G-712C [114] are other D3 receptor variations that have demonstrated a potential relationship with schizophrenia that some consider to be relatively nominal. Indeed, studies are yet to demonstrate a truly extensive association with schizophrenia [115, 116]. Other studies still have evaluated the relationship between polymorphism and the response to the atypical antipsychotic clozapine [24] which was observed, but not in all studies [117, 118].

### 4.2. Serotonergic Receptors Coupled to G Protein and Schizophrenia

Although there is contradictory evidence that serotonin receptors are changed in schizophrenia, pharmacological results of the serotonergic action of propsychotic drugs of abuse and atypical antipsychotic medications show that serotonin plays a crucial role in such illnesses. The strongest evidence for serotonin's significance in schizophrenia has come from pharmacological studies. D-Lysergic acid diethylamide (LSD) can elicit psychotic symptoms in healthy people because of its structural similarity to serotonin, prompting further inquiry into this neurotransmitter system in psychosis. LSD affects the raphe nucleus' serotonin system via 5HT1A receptors, and hallucinations are almost certainly induced by the agonist activity of the 5HT2A receptor [119]. The last two decades of research described at least fifteen 5-HT receptor subtypes based on the specific biochemical signaling pathways, as presented in Table 1.

### 4.3. Glutamatergic Receptors Coupled to G Protein and Schizophrenia

A great deal of evidence suggests that the glutamatergic system is involved in schizophrenia. The majority of the existing evidence, as with mood disorders, is focused on the role of ionotropic glutamatergic receptors. There are several reviews that cover this topic [121, 122]. Support of a glutamate role in schizophrenia is centered largely on pharmacological investigations. Studies have shown that phencyclidine (PCP) and amphetamine-instigated schizophrenia-like social symptoms are potentiated by group-I enemies of metabotropic glutamate receptors (mGluR1 and mGluR5) [123]. Get-together, group-I agonists limit sensorimotor gating impairments mediated by amphetamine and PCP-induced dopamine discharge in rodents in the prefrontal cortex [124]. Agonists of the get-together II receptor (mGluR2/3) block direct incitement achieved by PCP and working memory impairment [125, 126]. In sensorimotor gating, which aggravated schizophrenia, knockout mice mGluR1 and mGluR5 exhibit exacerbation [123].

## 5. GPCRS in Depression

### 5.1. Depression and Antidepressant Activity G Protein Subunits

The majority of the current antidepressant medications interact with G protein-coupled receptors (GPCRs). For example, 5-HT1A receptor acting partial agonists buspirone or aripiprazole mediates their effects through a variety of monoaminergic GPCRs, thereby affecting endogenous synapse levels. They produce serotonin reuptake inhibitors (SSRIs) like fluoxetine and monoamine oxidase inhibitors (MAOIs) like selegiline, as well as serotonin reuptake inhibitors (SSRIs). Members of an intracellular family of heterotrimeric G proteins, consisting of a G*α* subunit and a *βγ* dimer, form a multiprotein complex with seven transmembrane domains with G protein-linked receptors [54].

#### 5.1.1. Levels of G Protein Expression

Antidepressant drug therapy does not appear to alter G protein expression levels in the central nervous system (CNS) consistently in preclinical trials. Chronic treatment with the tricyclic antidepressant imipramine results in relatively stable mRNA expression of G*α*s, G*α*o, and G*α*i in the rat hippocampus, according to one study [127]. However, the measurement of G protein mRNA levels does not necessarily provide insight into its protein expression [128]. As a result, the amount of G protein present is not always accurately reflected in these observations. In studies focused on protein level measurements, those of G*α*s, G*α*o, and G*α*i in the rat cerebral cortex were unaffected by chronic treatment with amitriptyline, a dual serotonin-norepinephrine reuptake inhibitor (SNRI), or by desipramine, tranylcypromine, or electroconvulsive shock [54, 129].

#### 5.1.2. Impact on G*α*s Localization and Signaling

Notwithstanding the absence of any immediate consequence on G protein verbalization levels, treatment with solutions of energizers (checking for amitriptyline, desipramine, and iprindole) brings cAMP focuses up in the brain of rodents, not in the liver or kidney, in a GAPS-subordinate manner [130–132]. Notwithstanding energizer arrangement items, constant electroconvulsive treatment reinforces the coupling among GHAs and adenyl cyclase. Also, extended cAMP-subordinate kinase movement (for example, protein kinase A) was seen in the rodent cerebrum after predictable upper treatment, reliable with this all-encompassing creation of adenylyl cyclase [133]. When desmethylimipramine was led constructively, but not brutally, these modifications in the cerebral cortex were not observed in the hippocampus, striatum, or cerebellum [134].

## 6. G Protein Signaling Regulators as a Potential Drug Target for the Central Nervous System (CNS)

G protein-coupled receptors (GPCRs) are colossal focuses for medication exposure. In the transduction of GPCR signals, the G protein signaling- (RGS-) protein family controller has a primary breaking point. GPCRs are common pharmacological targets. RGS proteins quicken the deactivation of G proteins. Likewise, they produce communication signals to diminish GPCR flagging. The blend of GPCR agonists with inhibitors of RGS could potentiate reactions and could essentially expand the geographic particularity of the agonist. Because of their diversity and highly localized and dynamically regulated distributions in the brain, RGS proteins are intriguing targets for the pharmacotherapy of central nervous system illnesses [135].

### 6.1. The Dual Role of RGS Proteins as Either Inhibitors Or Effectors in GPCR Signaling

(a) RGS proteins such as G*α* GTPase accelerating proteins (GAPs): the binding of agonists to G protein-coupled receptors (GPCRs) induces the receptor guanine nucleotide-trade (GEF) action, leading the loading of GTP by the G*α* subunit, conformational changes in the G*α* switch areas (I, II, and III), making the dynamic ∗*α*-accreditation, separation of the G*α*–G*βγ* complex, and resultant effector interactions. RGS proteins decrease GPCR motioning by inducing the speed of GTP hydrolysis by the G protein *α*-subunit, which prompts G*α*–G*βγ* reassociation. Blocking the binding of the RGS-box to G*α*•GTP, for the current condition, would induce a delayed lifetime of the G*α* subunit in the GTP-bound state, improving the receptor-engaged reaction through elevated levels of free G*α*•GTP and G*βγ* subunits. (b) RGS proteins as G*α* effectors: RGS proteins, in a similar way, have a positive occupation in GPCR motioning as a result of p115-RhoGEF and the related proteins PDZ-RhoGEF and LARG. On binding, G13*α*, the GEF action of p115-RhoGEF broadened, affecting RhoA incitation, accomplishes the downstream impacts of cytoskeletal changes and transcriptional control. P115-RhoGEF additionally deactivates G13*α* by procedures for RGS-box GAP improvement, at any rate not before the signal is transmitted forwards. For the current situation, inhibiting the RGS-box–G*α* interactions by small particle mediation should decrease G13*α*-subordinate RhoA beginning. LARG stands for leukemia-associated RhoGEF; Pi stands for phosphate group; RGS stands for the regulator of G protein signaling [135].

### 6.2. Role of Endogenous RGS Protein

The dormant synaptic potential formed when heterotrimeric G proteins change molecule channels are signaled by metabotropic G protein-coupled receptors. Numerous neurons create excitatory postsynaptic possibilities interceded by G proteins of the G*α*q/11 family, thus enacting phospholipase C-*β*. GTPase-actuating proteins (GAPs) are believed to be needed to quicken GTP hydrolysis and quickly turn off G proteins. However, the contribution of GAPs in neuronal G*α*q/11 flagging has not been analyzed. It has been shown that controllers of G protein flagging (RGS) proteins provide a vital GAP movement at neuronal G*α*q/11 subunits. Obstruction of neighbourhood 2-pore region potassium coordinates in cerebellar granule neurons was reconstituted by conveying fanciful G*α* subunits that are activated by G*α*i/o-coupled receptors, in this way bypassing endogenous G*α*q/11 subunits [136, 137] .

### 6.3. Specificity of RGS Protein

RGS proteins are common negative G protein signaling regulators that work by speeding up the rate of GTP hydrolysis on G protein *α* subunits [138]. The large number of signaling pathways involving RGS and G proteins raises the question of how specificity in mutual recognition is achieved. In examinations using scrubbed proteins or proteins conveyed in cell culture [135, 139, 140], progression towards this way is made by portraying the instances of unique RGS-G protein associations. To fully comprehend the specificity of RGS protein function within a given G protein cascade, however, this knowledge is insufficient on its own. RGS proteins may also be needed to distinguish between the free activated G protein *α* subunits and their complexes with effectors or other regulatory proteins in order to make sure that the timing of the full signaling event delivered by a certain route is physiologically acceptable [141].

### 6.4. RGS Proteins as Drug Targets

Numerous neurological diseases are linked to RGS proteins. The development of RGS-insensitive G*α* subunits and RGS-insensitive knock-in mice has greatly contributed to our knowledge of the role RGS proteins play in these disorders [142]. These mutant G*α* subunits cause an uncoupled RGS-G*α* state, which is critical to understanding the implications of disrupting this interaction on the body's physiological functions. RGS proteins play a critical role in signal transduction, as demonstrated by subsequent RGS deletion or knockdown studies that replicate an environment in which GPCR signaling is not restrained by RGS proteins [143–145].

### 6.5. RGS Inhibitors as Clinical Therapeutic Agents

RGS proteins, which offer an alternate approach of controlling the activity of G protein-coupled receptors, the target of many medications, have recently come to light as possible therapeutic targets. RGS protein inhibitors must be able to cross the blood-brain barrier, be permeable to cells, and disrupt protein-protein interactions (RGS-G*α*), depending on the therapeutic target [135].

## 7. GPCRS in CNS

Extensive examinations of the genome sequence databases of humans and mice have revealed that the nonodorant GPCR superfamily consists of 392 receptors in mice and 367 receptors in humans, with 343 receptors being mutually expressed between these two species. Within the CNS, nonodorant GPCRs are abundantly expressed, with 6 clusters being noted [17, 146].

### 7.1. Medium-Sized Spiny Neurons (MSNs) Control Psychiatric Symptoms in the Striatum

The striatum is the basal ganglia's main input structure, and dopamine primarily regulates how information is processed there [147]. The nigra pars compacta (SNc) and the ventral tegmental area (VTA), containing nuclei associated with reward processing, reinforcement learning, and motor control, both innervate the striatum, with dysfunction in the VTA, in particular, being associated with schizophrenia. Within the striatum, MSNs, which represent a specialized type of GABAergic inhibitory cells, represent some 95% of neurons [148, 149]. MSNs have striatonigral and striatopallidal pathways and, based on their projection, can be classified into two types of neuronal populations. The striatonigral (direct) pathways project onto the medial globus pallidus (MGP) and the SNr (substantia nigra pars reticulate) and express dopamine D1 receptors and neuropeptide substance P. Adenosine A2A receptors, neuropeptide encephalin, and dopamine D2 receptors can be found in the striatopallidal (indirect) pathways, which project onto the lateral globus pallidus (LGP) [150, 151]. In the light of the known involvement of MSNs in the development of schizophrenia and PD [147], their differential functions require elucidation.

### 7.2. Antipsychotics Exerts Therapeutic Action through Dopamine D2 and D1 Receptors

Dopamine D2 and Gs-coupled dopamine D1 receptors are both strongly expressed in the striatum, which is where the majority of routinely prescribed antipsychotics are taken [147]. Schizophrenia has complex inheritance patterns and includes cognitive deficits and positive and negative symptoms [152]. These schizophrenic symptoms indicate a hyperactive dopaminergic transmission (mesolimbic pathway) as well as a lessened dopamine release in the prefrontal cortex [153]. Aripiprazole is a partial dopamine D2 receptor agonist and is classified as an atypical antipsychotic. In a situation of excessive dopaminergic neurotransmission, it also functions as a D2 receptor antagonist [154]. In schizophrenia, D2 signaling is considerably hyperactive [155], and in the prefrontal cortex, the D1 receptor is decreased [156].

### 7.3. Striatal-Enriched GPCRs Are a Potential Drug Target for Psychiatric Disorders

**Figure 2:**
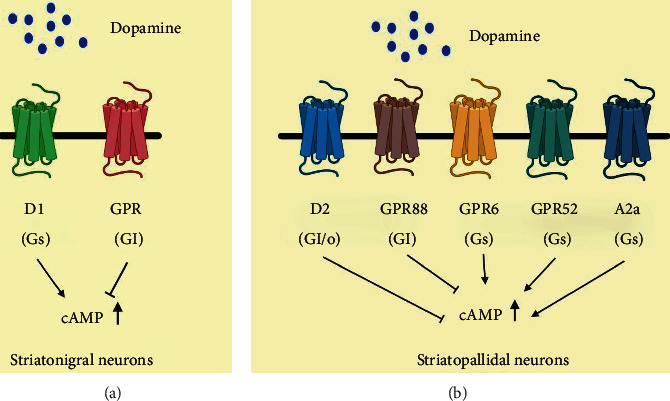
GPCRs expressed in the striatum in medium-sized sharp neurons (MSNs). MSNs are isolated into two primary categories: striatonigral (a) and striatopallidal (b) neurons.

The striatum is an important part of the brain that might be studied as a possible treatment target for mental disorders. GPR88, GPR52, and GPR6, as well as dopamine D1 and D2 receptors and adenosine A2a receptors, are the most highly expressed GPCRs in the mouse striatum, according to transcriptional analyses [17]. GPR88, GPR52, and GPR6 are orphan GPCRs, and their transcriptional expression patterns are almost indistinguishable from D2 and D1 receptors in the cerebrum. In both striatonigral, GPR88 is communicated, and striatopallidal neurons communicate D2 and D1 receptors, individually, as per in situ hybridization (ISH) investigation (basal ganglia), while GPR6 and GPR52 are communicated in the striatopallidal neurons (Figure 2). Within the taking after segment, these three vagrant GPCRs, as well as the A2a receptor, are briefly clarified.

#### 7.3.1. Adenosine A2a Receptor

Adenosine regulates a wide range of brain activities by acting on adenosine A1 and A2a receptors. The A2a receptor is reported to have a major impact on a variety of neuropsychiatric functions, fundamentally through glutamatergic and dopaminergic neurotransmission, which may have potential value in a number of neurological disorders. Niemann-Pick disease, autism-spectrum disorders, and schizophrenia have been linked to A2A receptor agonists, whereas A2A receptor antagonists have been linked to Alzheimer's disease, attention deficit hyperactivity disorder, fragile X syndrome, depression, and anxiety [9, 157]. The Gs-coupled A2a receptor is preferentially expressed in the striatopallidal MSNs (Figure 2) and is reported to play a key role in motor function regulation as its ligand can trigger characteristic motor effects [158]. In this light, GABAergic communication from striatopallidal MSNs guided by A2a receptor antagonists may provide a PD treatment strategy [159, 160]. As the A2a receptor can regulate dopaminergic neurotransmission, this may, hence, have psychopharmacological implications. A key component that is considered to allow A2a inhibitors to intervene in motor activity is by modulating GABA discharge.

Some researchers believe that future treatments for mental illness will be based on an inhibitory interaction between D2 and A2a receptors. Antipsychotic effects of psychostimulants can be altered by A2a agonists in animal models [161]. Pharmacological and hereditary investigations show that the movement of the A2a receptor impacts patients' schizophrenia-like behavior. Caffeine, as a nonselective A1 and A2a receptor antagonist, has demonstrated both positive and negative effects in schizophrenia, depending on when it is administered, for how long and when its actions are evaluated [162]. Most schizophrenia symptoms primarily derive from perturbed dopaminergic and glutamatergic neurotransmission; however, alterations in the adenosinergic system also have been reported. As the adenosinergic system is associated with motivational and cognitive processes, the impact of adenosine on dopaminergic- and glutamatergic-mediated neurotransmission likely depends on the baseline status of these processes when the adenosinergic system is being manipulated. As noted, adenosine A1 and A2A receptor agonists reverse both NMDA receptor hypo- and dopaminergic hyperactivity. Whereas caffeine may worsen the positive symptoms of schizophrenia subjects (such as delusions and hallucination), a number of studies (but not all) have reported that it positively impacts learning and memory tasks [162]. However, cognitive performance can be affected by multiple potentially competing factors including attention and motivation as well as stress, and differential effects on these can well account for overall improvements in learning and memory under some conditions and worsening under others. Cellular investigations have demonstrated that adenosine A_2A_ receptor activation decreases the affinity of dopamine D_2_ receptor agonist binding sites on striatal neurons, without altering the affinity of dopamine D_2_ receptor antagonist binding or the expression of D_2_ receptors. Extensive studies reviewed by Ferré and colleagues support the concept that GPCR oligomerization can readily occur and that GPCR homodimers represent both functional and structural building blocks whereby heteromers, comprising of two different homodimers, can be formed that are each able to signal via their chosen G protein. This appears to be particularly likely in relation to A_2A_ receptor-D_2_ receptor heteromers and potentially accounts for their known allosteric mechanisms and multiple unique pharmacological and biochemical properties. On chromosome 22q12–13, a single nucleotide polymorphism (SNP) on the A2a receptor provides a candidate schizophrenia susceptibility gene [163], which, again, ties the adenosine A2a receptor to various mental disorders that include schizophrenia, as well as depression and anxiety. As indicated by a variety of studies, the adenosine A2a receptor “fine tunes” glutamatergic and dopaminergic network balance [164]. In this regard and as noted, the opposing interaction of D2 and A2a receptors in the striatum, in relation to dopaminergic action, provides potential antipsychotic activities in schizophrenia, as an antagonist of the dopamine receptor [161]. In relation to glutamatergic action and as reviewed by Komatsu [165], both A2a and A1 receptor agonists have been reported to reduce the electroencephalogram (EEG) and behavioral effects of NMDA receptor inhibitors [166]. It has been shown that NMDA receptor activity may be influenced by both A1 and A2A receptor function in schizophrenia models of NMDA receptor hypofunction. While the NMDA antagonist effects in animal models of schizophrenia are reversed by the blockage of A2a receptors and the genetic deletion of the receptor, this suggests that correcting the imbalance in adenosine A2a receptors may rectify the hypofunction of NMDA antagonists [167].

#### 7.3.2. GPR88

Human gene association studies show a rising correlation between GPR88 function and mental health issues, including schizophrenia and bipolar disorder as well as neurodevelopmental and neurodegenerative disease [168–170]. Following exposure to a variety of psychoactive substances, including antidepressants, mood stabilizers, and drugs of abuse, mRNA levels of Gpr88 have been observed to vary in animal models. Hence, in this light, GPR88 may provide a promising target in the development of new treatment strategies for neurological disorders. By deleting the Gpr88 gene in mice, striatal-dependent physiology, neural networks, and behaviors are affected, resulting in hyperactivity, stereotypies, and deficits in learning and motor coordination, as well as changes in reward-driven behaviors. The expression of GPR88 beyond the basal ganglia and its knockout suggest that GPR88 has potential influence on a broad range of behaviors and brain physiology; however, how this is specifically achieved from a molecular basis remains to be elucidated as, currently, selective pharmacological probes to manipulate GPR88 remain relatively few. Available structural studies suggest that GPR88 is an atypical GPCR. Although considered as part of the class A (rhodopsin-like) family of GPCRs, which is the largest group and accounts for approx. 80% of GPCRs (including hormones, neurotransmitters, and light receptors), GPR88 minimally shares amino acid sequence and functional similarities to other members of this class. So far as we know, the structure of the GPR88 transmembrane binding pocket is more in line with class C GPCRs (which include the metabotropic glutamate family, GABA receptors, calcium-sensing receptors, and taste receptors). Although significant efforts have been made to deorphanize GPR88 (i.e., discover ligands that are highly selective), several hurdles persist, and hence, the signaling pathway(s) and receptor capabilities of GPR88 remain mostly unknown. Studies including the electrophysiological evaluation of MSNs taken from knockout mice lowered tonic GABAergic inhibition and responses to synaptically generated GABA, whereas glutamatergic excitatory synaptic transmission was increased. This shows that GPR88 may play a role in glutamatergic transmission, together with the phosphorylation of the AMPA-type glutamate receptor GluR1 in GPR88 knockout mice. According to the findings, GPR88 appears to have a role in the response of GPR88 knockout mice to agonists of the muscarinic and opioid delta and mu receptors. GPR88 has been discovered as a genetic risk factor for bipolar illness and schizophrenia by genetic association analysis. GRP88 dimerization with nonorphan GPCRs is supported by recent findings [171–174] and demonstrates an ability of GPR88 to dampen the signaling of many GPCR receptors in close proximity as well as impede *β*-arrestin recruitment. As ligands for GPR88 are recently being described, including 2-PCCA, RTI-13951–33, and phenylglycinol derivatives, our understanding of the signaling pathways associated with GPR88 and its physiological roles will likely become clearer in the near future.

#### 7.3.3. GPR6

GPR6 is mostly expressed in the basal ganglia's striatopallidal neurons (Figure 2) [17]. GPR6 is an orphan receptor that has a ubiquitous function and generates an increase in intracellular cAMP levels when it is linked to a stimulatory Gs-protein. GPR6 was initially described as a lysophospholipid sphingosine 1-phosphate (S1P) receptor; however, this view has not been confirmed by others [175]. The corticostriatal circuitry and the dopaminergic system are considered engaged with human instrumental learning [176]. In rodent essential cerebellar granule neurons, overexpression of GPR6 augments neurite outgrowth [177]. Nishi and Shuto examined the neurochemical and behavioral phenotypes of GPR6 knockout mice to aid define the receptor's function. GPR6 deletion-mice demonstrated a decrease in striatal cAMP and an elevation in dopamine, as well as increased movement actions. A reduction in dyskinesia in a PD mouse model after therapy with apomorphine and quinpirole was also noted, implying that GPR6 inhibition may provide benefit PD. In GPR6 knockout animals, the phosphorylation of dopamine and the cAMP-regulated phosphoprotein of 32 kDa (DARPP-32) at Thr34 increased significantly, while the production of DARPP-32 in the striatum did not. The cAMP/PKA pathway phosphorylates DARPP-32 at Thr34, which is essential for the signal transduction of dopamine D2 and D1 receptors [178]. The D2 receptor antagonist haloperidol has been shown to increase DARPP-32 phosphorylation at Thr34 in striatopallidal neurons [178–180]. Increasing evidence supports the concept that GPR6 could potentially be targeted as a treatment strategy for schizophrenia.

#### 7.3.4. GPR52

**Figure 3:**
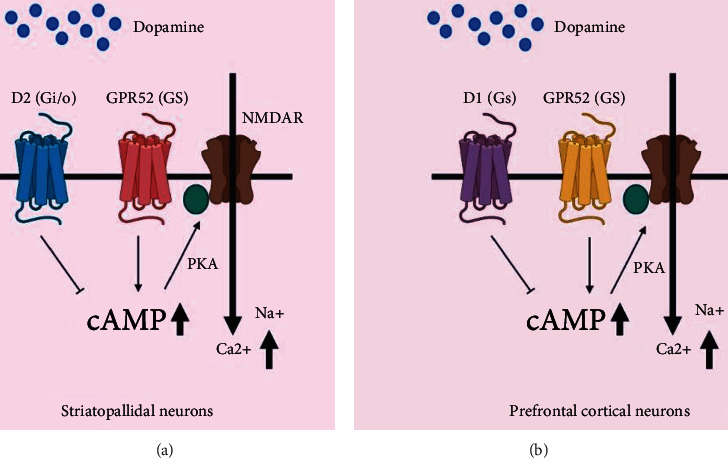
A potential sign transduction pathway for GPR52.

Despite the fact that the antipsychotic medicine reserpine is reported to be a surrogate ligand for actuating intracellular cAMP accumulating and receptor internalization, GPR52 is an orphan GPCR, indicating that it may be a Gs-coupled receptor [165]. Over 90% of the amino acid corrosive grouping property of GPR52 is conserved in vertebrates, and it is broadly distributed in the brain, notably the striatum, with no apparent alterations between species. Despite the fact that GPR52 is expressed in the basal ganglia, it also communicates with a high number of D1-expressing neurons in the prefrontal cortex. This is an unusual expression profile (Figures 2 and 3). As a result of this, GPR52 activation may promote good effects of schizophrenia by antagonizing Gi/o-coupled D2 receptor action in striatopallidal MSNs, while also reducing schizophrenic adverse effects and cognitive disability by upgrading NMDA receptor movement in prefrontal cortical neurons via protein kinase A (PKA), as can be seen in D1 receptor-NMDA signal transduction [181, 182].

An extensive study of GPR52-expressing neurons' axonal extensions shows that the limbic neural circuit, which is critical for memory and spatial memory, is communicated by GPR52. Dopaminergic neurons in the midbrain get a negative compensatory signal from GPR52-expressing neurons in the habenular nucleus [183]. Prefrontal cortex GPR52-expressing neurons are almost entirely glutamatergic, while only around 10% are GABAergic. The GFP-fused human GPR52 is useful in vitro in hGPR52-GFP transgenic (Tg) mice, and hGPR52-GFP and D2 receptor proteins are clearly separated across striatal regions. The GPR52-GFP is located largely in the LGP, while the bulk of D2 receptor proteins are detected in the striatum. LGP's axon terminals contain GPR52, whereas the dendritic spines of striatal neurons are home to D2 [17]. Up until now, GPR52 transgenic (hGPR52) and GPR52 knockout (KO) mice seemed to be created, as well as examined. The methamphetamine- (MAP-) interceded hyperlocomotion of hGPR52 Tg mice is less articulated than non-Tg mice, despite how hGPR52 Tg mice have ordinary locomotor action under typical conditions, showing that overexpression of GPR52 can neutralize hyperdopaminergic transmission prompted by MAP. GPR52 KO mice spend more time in the center zone of the open field test, indicating that they have anxiolytic-like behavior. NMDA receptor antagonist MK-801 makes GPR52 KO mice more susceptible to the startle reaction of the induced hindrance test (PPI). The startle reflex's habituation and PPI are slowed in schizophrenia patients. However, both conventional and atypical antipsychotics enhance PPI deficiencies and reduce startle responsiveness [184]. These data demonstrate that GPR52 appears to affect not just dopamine transmission but also NMDA signaling [185]. An intense, orally accessible GPR52 agonist with excellent pharmacokinetic properties was recently developed. After the oral administration of 3 mg/kg of methamphetamine, this agonist generously diminishes methamphetamine-instigated hyperactivity in mice and has reported extrapyramidal indications (EPS), proposing that GPR52 enactment checks effective dopaminergic transmission in the limbic framework. The disclosure of GPR52's atomic component could prepare for a new examination for the dopamine and NMDA frameworks, just as the advancement of new antipsychotic drugs. Some GPCRs in the specific types of psychiatric disorders with the function, polymorphisms, and drugs targeting receptors are shown in Table 2.

## 8. Treatment of Psychiatric Disorders

### 8.1. Modes of Action of Antipsychotics in Psychiatric Disorders

Catecholamine neurotransmitters, such as dopamine, are found in the brain [186]. Parkinson's disease, schizophrenia, obsessive-compulsive disorder, bipolar disorder, and hyperactivity-hyperactivity disorder are only a few of the CNS illnesses where dopamine plays a major role [187, 188]. D1 and D2 dopamine receptors are two subclasses of G protein-coupled receptors (GPCRs), which are responsible for the action of dopamine. When it comes to receptor subclasses, D2 has D2R, whereas D1 has the D1 receptor (D1R), while receptor subclasses D5 and D2 and subclasses D4 and D3 each have their specific receptor (D5R) [189]. The basal ganglia receive their information mostly from the striatum, which has the greatest number of D1R and D2R neurons [190]. In schizophrenia, the SNc and the VTA both have a substantial impact on striatum innervation; it is considered that the VTA is hyperactive in positive symptoms [191]. The striatum includes 5% of interneurons including large aspiny cholinergic neurons and 95% gamma-amino butyric acid (GABAergic) medium-sized spiny neurons (MSNs) [151], composed of direct (striatonigral) and indirect (striatopallidal) pathways [192]. The striatonigral neurons transmit onto the MGP (medial globus pallidus) and SNr (substantia nigra pars reticulate) and prompt D1R and P, neuropeptide substances. The striatopallidal neurons project toward the globus pallidus (LPG) and contact the SNr/MGP. Moreover, it can express D2R, A2a adenosine receptor, neuropeptide encephalin, and two orphans GPCR, GPR6 and GPR52. The direct and indirect pathways regulate motor behavior by smoothing locomotion and revoking movements [153, 193, 194].

**Figure 4:**
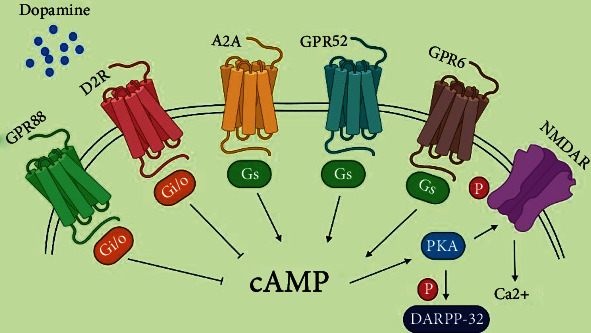
Model of GPCR-mediated NMDA receptor pathways in striatopallidal MSNs by phosphorylating it with cAMP.

**Figure 5:**
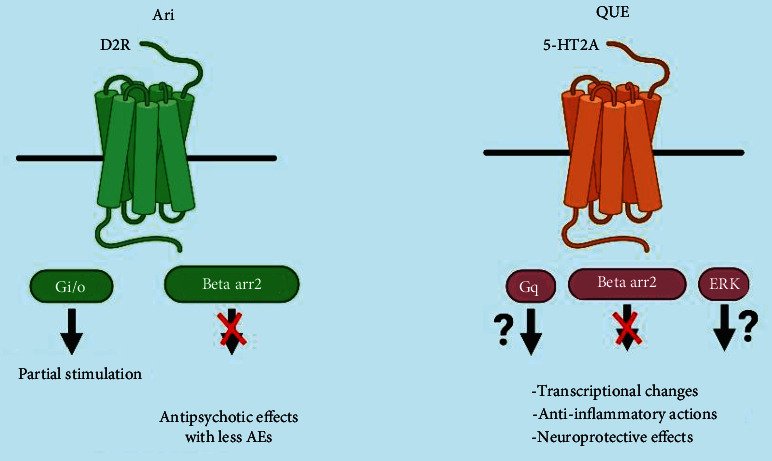
Proposed antipsychotic actions to confer bias of GPCR signalling.

Reactivation of D2R stimulates Gi/o-facilitated signalling in striatopallidal neurons, which inhibits the PKA (protein kinase A)/dopamine and cAMP-regulated phosphoprotein of 32 (DARPP-32) kDa channel, which can help with many dopamine-related behaviors [152]. Some other examples that can also regulate this pathway are Gs-coupled GPR52, GPR6, Gi-coupled GPR88, and adenosine A2a (Figure 4). These GPRs Gs-coupled are helpful to enrich NMDA (N-methyl-D-aspartate) receptor activity via cAMP/PKA. Dopamine shows a remarkably attenuated response to amphetamine in mice lacking *β*arr2. A protein-protein interface occurs in D2R, and D1SC1 is found as an inducer of hyperactive activities in mice that needs to signal *β*arr2 to promote phosphorylation of AKT and consequent activation of GSK3*β* [153]. The mood stabilizer lithium hinders the triggering of GSK3*β* and collaboration in *β*arr2 and AKT [21]. The initial mode of action of antipsychotics in basal ganglia is antagonism of D2Rs and the first-line treatment for bipolar disorder and schizophrenia. As a result, a genome-wide association study (GWAS) discovered the gene D2R within a schizophrenia-associated region to be effective for etiology and therapy [195], although antipsychotic medicines are not medically practicable for reducing cortical-related symptoms [196]. The classification of antipsychotics is typical and atypical. Haloperidol is a typical antipsychotic (first-generation antipsychotics) which principally has an inhibitory activity for D2Rs. Quetiapine, risperidone, and some others are examples of atypical antipsychotics (second-generation antipsychotics). They have an antagonistic activity for D2Rs, 5-HT2A (serotonin 2A receptor), and other several GPCRs, and they have fewer side effects than typical antipsychotics (Figure 5).

Different investigations have argued that a hyperdopaminergic state is the only reason for the development of schizophrenia. Additional evidence shows that the hyperdopaminergic state in the basal ganglia and the hyperdopaminergic state in the frontal cortex are also responsible (according to the dopamine hypothesis). Based on the reorganized dopamine hypothesis of psychosis [122], antipsychotics that block D2Rs were thought to simply reverse striatal hyperdopaminergic activity. So, the antipsychotic drug should simultaneously activate and inhibit dopamine signalling according to the brain region [197]. Antipsychotic medication might benefit from the discovery that D2Rs are capable of signaling not just through standard G protein pathways but also through noncanonical pathways that induce the creation of a signaling complex involving protein phosphatase2 (PP2A), GSK3*β*, 5AKT, and *β*arr2. Haloperidol initiates AKT phosphorylation in the brains of mice that could recompense for the defective activities of *β*arr2-GSK3*β* pathways in schizophrenia. D2R-arr2 recruitment is blocked by all clinically effective antipsychotic medications. Antipsychotics which mainly target the pathway of D2R-*β*arr2 might have more therapeutically favourable effects but have fewer side effect like EPS [21, 178]. BRD5814, an arr2-biased D2R antagonist, improves dopamine-induced hyperlocomotion in mice while reducing motoric side effects [198]. Hence, the mechanisms of quetiapine and aripiprazole which are frequently used as antipsychotic drugs are discussed in the following sections.

#### 8.1.1. Aripiprazole

Aripiprazole is an antipsychotic medication that was first approved for the treatment of a neuropsychiatric disorder called schizophrenia, which affects more than 1% population of the world. At D2R-Gi/o pathways, aripiprazole works as a partial agonist beneath high dopaminergic tone. They act like a biased D2R-Gi/o inhibitor [199]. Aripiprazole's agonist activity can completely block the D2R-arr2 translocation, which is triggered by dopamine. Aripiprazole also has antagonistic effects on other antipsychotics, such as hyperprolactinemia, metabolic disorder, extrapyramidal symptoms (EPS), weight gain, and sedation [20]. Aripiprazole does not affect the progression of cognitive impairment in schizophrenia patients. Parvalbumin, a calcium-binding protein, and mRNA transcription of the GABA-producing enzyme glutamic acid decarboxylase 67 (GAD67) are both downregulated in an autopsy of a patient with schizophrenia [200]. D2Rs are mediated in GABAergic FSIs in the prefrontal cortex (PFC), which control the activity possibilities of glutamatergic pyramidal neurons. Aripiprazole is only a partial agonist of D2R-Barr2 in the presence of GPCR kinase 2 (GRK2) in the cortical FSIs, although it has a limited effect on action potential firing [201]. Therefore, aripiprazole completely blocks the D2R-*β*arr2 pathway whereas stabilizing the D2R-Gi/o channel in the hyperdopaminergic striatum of the syndrome.

#### 8.1.2. Quetiapine

Quetiapine, a typical antipsychotic agent, has been approved for diseases like schizophrenia and bipolar disorder [64]. Metabolic nor-quetiapine may mediate the antidepressant action of quetiapine, at least in part, via noradrenaline transporter inhibition [202]. Patients with bipolar illness have been found to benefit from immediate-release (IR) and extended-release tablets (XR) of quetiapine, according to several studies [203]. Further investigation uncovers that a patient with schizophrenia has abnormal cytokine expression. Quetiapine shows unique anti-inflammatory and neuroprotective properties [107]. Quetiapine reduces the activation of astrocytes and microglia, as well as the synthesis and release of two cytokines, TNF-alpha and MCP-1, in mice (MCP-1). These results recommend that quetiapine may block injury by releasing cytokines and inhibiting glial cells' neuroinflammatory response. Quetiapine also shows activity by neuroprotective effect by increasing the deliverance of brain-derived neuroprotective factor (BDNF) in contradiction of amyloid toxicity from cultured astrocytes [204]. Quetiapine shows unique regional and temporal transportation of epidermal growth factor receptor (EGFR) channel in mice, exhibiting EGFR-dependent striatal ERK1 activation and cortical ERK1 phosphorylation via aripiprazole is EGFR autonomous. The molecular actions of quetiapine are composite and could include influenced signaling [205].

## 9. Drug Discovery Areas for Psychiatric Disorders

Drug discovery for psychiatric disorders is slow and tedious [206]. There are several pathophysiological processes associated with genetic and environmental factors that may obstruct the drug development of such diseases [206, 207]. One of the major areas is target validation which contributes to the development of new drugs. Human brains are capable of storing a large amount of data, which can render our brains vulnerable in response to a stressful experience. For instance, PTSD is a psychiatric disease where patients can memorize horrible experiences like rape from their entire life. Trauma coupled with remembering past events activates neural plasticity in this situation [208]. Dysregulation of neuronal plasticity and brain atrophy is responsible for stress-related psychiatric disorders. Therefore, it has been suggested that the drug which possesses inhibitory effects on neuronal plasticity will be helpful in the management of this disorder [206]. Stress and depression are caused by glutamate hyperactivation, which is controlled by presynaptic proteins such as CaM kinase II. As a result, it may provide an opportunity to develop novel antidepressant medications [209]. Neuroimaging can provide an exact diagnosis of disease as well as the location of brain dysfunction. Developing new drugs will be feasible when the diagnosis is specific [206]. Additionally, imaging-based diagnosis has been an aid to the clinical management of psychiatric disorders.

## 10. Conclusions and Future Perspectives

Neurotransmitter imbalance leads to brain dysfunction in people with psychiatric illnesses. The GPCR may have a role in these conditions, as well as in psychiatric difficulties. As a result, the creation of new treatment options and pharmacological tactics is made easier with an in-depth knowledge of G protein-coupled receptor activities and signaling pathways. Several studies suggest that this dysfunction of ROS, glutathione, and oxidative stress is responsible for the alteration of brain function. The fact is that innovative drugs that simply imitate “traditional” treatments by altering neurotransmitter levels directly or indirectly may be of limited help to many patients with refractory illnesses. Altering synaptic activity will alter the system's postsynaptic “throughput,” because such tactics presuppose that target receptors and downstream signal mediators are functionally intact. The direct targeting of postreceptor sites may be the sole strategy to enhance therapy for patients who are resistant to traditional medications, given the likely existence of GPCR abnormalities (and perhaps their signal transduction pathways). Research into the generation and deactivation of second messengers may lead to novel pharmacological medicines. Developing novel treatments that target second messenger systems may be possible since they are exceedingly varied at the molecular and cellular level, are connected to receptors in several ways, and are expressed in a wide variety of cell types and tissues. This, on the other hand, is more difficult than the development of receptor-targeted medications. These sites on the substrate serve as built-in targets for relative specificity of action since signal transduction pathways show varied properties based on their active state. Very recent achievements in new medicines for the long-term management of these serious mental illnesses are hugely encouraging. When it comes to the next phase of neuropsychopharmacology, it will be critical to apply the information obtained from advances in neuroscience and pharmacology in clinical settings. Moreover, a number of compounds and strategies have been discussed in this review for managing mental illness. Further investigations are required to develop standard treatment strategies with effective neuropotential effects. The present treatment approaches should be observed carefully to ensure their efficacy, or it should go for further development. However, more in-depth studies are required to identify more effective treatment approaches that aid in the improvement of current treatment methods and strategies. GPCR expression data from mice and humans can be used to model GPCR function in mental illnesses in a manner that is appropriate for animals. This data may also be used to find new therapeutic targets and anticipate on-target side effects.

## Figures and Tables

**Table 1:** The family of 5-HT receptors [120].

Receptor	Selective agonists	Potential	Type	G protein effector	Mechanism of action	CNS distribution
5-HT1 (5-HT1A, 1B, 1D–F)	1A=8-OH-DPAT, 1B=sumatriptan, 1C=sumatriptan, 1F=LY 334370	Inhibitory	Gi/G0-protein coupled	Gi/o	Decrease in intracellular cAMP levels and inhibition of AC	Cerebral and frontal cortex, hippocampus

5-HT2 (5-HT2A–C)	DOI^d^, BW 723C86, Ro 600175	Excitatory	Gq11-protein coupled	Gq/11	Activation of PLC, enhancing in IP3 and DAG intracellular concentration, and rise in intracellular calcium	Nucleus accumbens, basal ganglia, cerebellum

5-HT3 (5-HT3A, 3B)	DOI^d^, BW 723C86, Ro 600175	Excitatory	Ligand-gated Na+/K+ channel	—	Depolarization of cell plasma membrane	Hippocampus, amygdala, nucleus accumbens

5-HT4 (5-HT4A–H)	DOI^d^, BW 723C86, Ro 600175	Excitatory	Gs-protein coupled	Gs	Increased intracellular cAMP concentration and activation of AC	Hippocampal membranes

5-HT5 (5-HT5A)	—	Inhibitory	Gi/G0-protein coupled	Gi/o	Decrease in intracellular cAMP levels and inhibition of AC	Olfactory bulb, neocortex, hippocampus

**Table 2:** Some GPCRs in the specific types of psychiatric disorders with the function, polymorphisms, and drugs targeting receptors.

Some GPCRs in psychiatric disorders	Normal function	Polymorphism/change in expression	Drugs targeting receptors
5HT1A receptors	Evidence that continuous antidepressant treatment results in desensitization of somatodendritic 5HT1A auto receptors in the dorsal raphe, and subsequent increase serotonergic transmission in the hippocampus supports the hypothesis that 5HT1A receptors play a role in mood disorders.	Five-HT1A receptor data from numerous research suggest that the 5HT1A receptor has a role in depression and treatment response. Lemonde and colleagues establish a link between the C-1019G 5HT1A promoter polymorphism and serious depression, suicide, and the effectiveness of the antidepressant flibanserin, a 5HT1A agonist.	Chronic lithium therapy in bipolar individuals normalizes abnormalities in 5HT1A receptor binding.

5HT2A receptors	It has been demonstrated that antidepressants decrease the cortex's 5HT2A receptor binding.	Five-HT2A receptor A-1438G, a promoter polymorphism in the 5HTR2A gene, has been linked to serious depression. There have been numerous attempts to find a link between 5HT2A receptor polymorphisms and bipolar disorder, but these studies have not consistently shown any.	Studies examining the binding of the 5HT2A receptor after chronic lithium medication have yielded conflicting findings. Although studies in platelets have shown that lithium-induced increases in 5HT2A receptor binding capacity in bipolar individuals, the majority of research imply that lithium generates a decrease in 5HT2 receptor binding, with the strongest evidence in the hippocampus.

## Data Availability

All data are available within the text.
